# Understanding the Public Policy of Global Budget Payment Reform Improves the Quality of Public Healthcare From the Perspective of Patients in China

**DOI:** 10.3389/fpsyg.2022.911197

**Published:** 2022-07-22

**Authors:** Lele Li, Shuo Zhang

**Affiliations:** ^1^School of Labor and Human Resources, Renmin University of China, Beijing, China; ^2^School of Economics and Management, Tsinghua University, Beijing, China

**Keywords:** retrospective payment system, prospective payment system, healthcare insurance payment method, global budget, the quality of public healthcare

## Abstract

The transformation from the retrospective to the prospective payment system is significant to improve the quality of public healthcare (QPH). This article used the quasi-natural experiment of the global budget payment reform of government (GBPRG) in Chengdu, adopted the difference in difference (DID) method to estimate the effect of the GBPRG on the QPH, and concluded that GBPRG has a significant positive impact on the healthcare outcome and has a significant effect on improving the QPH. Policy implications drawn from the results show that the government should continue to explore compound healthcare insurance payment method (HIPM), improve the governance capabilities of the government, reduce transaction costs, improve healthcare insurance reimbursement policies, adjust the proportion of healthcare insurance reimbursements, continuously optimize the allocation of healthcare resources, establish an incentive mechanism to improve the QPH, and realize the pareto optimal choice of healthcare resource allocation.

## Introduction

Before 1980, most countries in the world implemented the retrospective payment system, in which the providers of healthcare providers, such as medical institutions and doctors, played a leading role. As the retrospective payment system lacks cost restraint on both supply and demand sides of healthcare service, it has become an important reason for the rapid growth of medical costs in recent decades (Fan and Savedoff, 2014). Based on this background, the reform of healthcare insurance payment method (HIPM) has become one of the core contents of healthcare insurance reform all over the world. In the 1990s, along with the continuous advancement of welfare and healthcare reform in western countries, the responsibility and function of the healthcare insurance unceasingly strengthen. HIPM has gradually changed from a retrospective to a prospective payment system, while the reform direction has also developed from single cost control to equal emphasis on cost control and quality (Wang and Wu, 2017).

Since the global budget payment reform of government (GBPRG) was carried out in China, many researchers have conducted detailed studies on the process and current situation of the reform (Cai et al., 2013; Zhang and Ye, 2014; Yao et al., 2017), and established theoretical models and empirical analysis based on the effect of GBPRG on controlling healthcare expenses (Sun et al., 2012; Liu, 2014; Lang et al., 2017; Li and Chu, 2020). However, except for the effect on cost containment, studies have not reached much agreement on other impacts of the GBPRG (Chen and Fan, 2015), especially the impact of GBPRG on the quality of public healthcare (QPH).

This paper contributes to both the theoretical and empirical literature. This study extends the current theory by constructing the theoretical framework to reveal the influence mechanism of GBPRG on QPH. Second, previous studies either used healthcare record data or healthcare insurance settlement data to analyze the impact of GBPRG. This research creatively used computer science and big data technology to match the urban employee healthcare insurance settlement data from the government with healthcare record data from hospitals for the first time. Then, we used the quasi-natural experiment of GBPRG of Chengdu, adopted the difference in difference (DID) method to identify the effect and mechanism of GBPRG on QPH, and presented policy suggestions and optimization path.

## Literature Review

Sufficient studies have shown that different HIPMs have different incentives for healthcare service providers, thereby affecting healthcare expenses and QPH (Chiu, 1997; Ma and McGuire, 1997; Cutler and Zeckhauser, 2000; Newhouse, 2004; Aron-Dine et al., 2013). As the cost paid by healthcare insurance depends on the total cost charged by the healthcare service provider, optimizing the HIPM is helpful to control the healthcare expenses (Yip and Eggleston, 2001; Moreno-Serra and Wagstaff, 2010; Pauly et al., 2012; Miller and Babiarz, 2014).

A global budget (GB) allocates a fixed number of resources to the healthcare sector or system (rather than individuals or organizations) to control overall healthcare expenses and ensure reasonable and affordable healthcare services. GB is an auxiliary payment method that could be combined with any other payment method to form a scheme suitable for different backgrounds. The main difference between various schemes is the constraint mechanism of the budget ceiling of the healthcare system (Zeng et al., 2018). In general, there are three different constraint mechanisms, namely, price adjustment, capitated payments to the health plans, and limiting a provider’s budget. The first mechanism adopted by the German Emergency Department, Taiwan and some Canadian provinces ensures that the total expense is fixed and the price of services is adjusted according to the volume of services. The two latter mechanisms that are usually taken to implement GBPRG by China and the United States pay a fixed amount to a medical program or service provider, and the difference between the budget and actual expenditure is a profit or loss. The effect of GBPRG on reducing healthcare expenses has been strongly proved globally (Chulis, 1991; Docteur et al., 2004; Ji et al., 2011; Hu et al., 2019).

However, the conclusion of empirical research about the impact of GBPRG on QPH are not consistent. Some scholars argued that because the quality improvement was accompanied by cost growth, and an expenditure cap resulted in a lower quality, GBPRG had a negative impact on the QPH (Cutler, 1995; Mougeot and Naegelen, 2005; Tung and Chang, 2010; Chang et al., 2011; Chen and Fan, 2015; Gao, 2017). On the contrary, some studies found that QPH was improved after the implantation of GBPRG (Wolfe and Moran, 1993; Shahian et al., 2005), while some researchers stated that there was no significant change in the QPH (Louis et al., 1999; Cheng et al., 2012; Kan et al., 2014).

Previous studies have not reached much agreement on the impact of GB on the QPH, and most of the related studies are descriptive or do not take into account the counterfactuals in the effect estimation. Consequently, it is rather difficult to validate those findings and reach a definitive conclusion on the impact of GB on the QPH. Furthermore, few studies tried to reveal the influence mechanism of GB on the QPH. Under the background of the full implantation of GBPRG in China, the aim of this study is to clarify the actual effect and mechanism of the influence of GB on the QPH and to explore the strategies and management measures for improving QPH to avoid quality risks.

## Institutional Background and Theoretical Framework

### The Historical Evolution of Healthcare Insurance Payment Reform

Healthcare insurance payment method, as an institutional arrangement of healthcare insurance fund to provide financial reimbursement for healthcare service providers on behalf of the insured, plays an important economic leverage role in reducing the burden of patients, controlling healthcare expenses, regulating healthcare service behavior, and promoting the allocation of healthcare resources. The HIPM is developing with the establishment, reform, and perfection of the healthcare insurance system in China.

In the early stage of reform and opening-up, the Chinese government implemented free healthcare. To achieve the goal of cost control, many regions introduced expense contracts under GB. In 1997, healthcare insurance for urban workers combined social orchestration with personal accounts, established the restriction mechanism for both doctors and patients, and actively explored scientific and reasonable payment methods to control the unreasonable growth of healthcare expenses effectively. In 1999, the basic framework of HIPM is clarified by means of GB under the prospective payment system, fee-for-service, and service unit payment. In 2009, the central government was required to improve the payment system and explore the implementation of capitated payments, case-based payments, and prepaid GB payments while encouraging active exploration of negotiation mechanisms and payment reform between healthcare insurance agencies and medical service providers. In 2012, GB was required to be carried out in all coordination areas in 2 years. Meanwhile, the new rural cooperative medical scheme was promoted and diagnosis-related groups (DRGS) payment was encouraged. In 2016, the Chinese government was required to comprehensively promote GB, accelerate to promote the application of DRGS, and establish a compound payment method. Under the guidance of the above policies, the role of Chinese healthcare insurance has changed from the payer afterward to the strategic buyer while the payment method has changed from the post-payment system to the prospective payment system and from a single payment method to multiple payment methods after more than 20 years of development. At present, HIPM has basically formed an overall framework based on GB, centered on consultation and risk-sharing mechanism, characterized by capitation payment for outpatient service, and case-based payment for a chronic and serious illness for outpatient and hospitalization, while the proportion of fee-for-service constantly decreases and the proportion of DIP and DRGS payment gradually increases.

### The Influence Mechanism of Global Budget Payment Reform of Government on Quality of Public Healthcare

Healthcare insurance payment method is an important part of basic healthcare insurance management and an important lever to regulate healthcare service behavior and guide medical resource allocation. As a social game rule, the institution determines the incentives faced by behavioral agents in the economy. A good system can reduce market transaction costs and improve resource allocation efficiency (North and Thomas, 1973). How to reform the payment system has become the most important problem of the new healthcare reform. This article focused on the following three aspects, specifically expounded on the theoretical mechanism of GBPRG affecting QPH.

#### Promoting Medical Service Information Disclosure and Reducing Information Asymmetry

From the perspective of principal–agent, the two parties constitute the simplest principal–agent relationship when only patients and hospitals participate in healthcare behavior. In this relationship, the patient is the client who entrusts the hospital (or doctor) to diagnose and treat the disease, while the hospital is the agent who accepts the entrustment of patients and chooses the treatment plan. Because of information asymmetry in healthcare services, hospitals will give unnecessary treatments to patients because of financial incentives.

The response to this problem is to increase client awareness of agents’ behaviors and their practical utility through monitoring, and reduce the degree of information asymmetry (Holmström, 1979; Hart and Holmström, 1987). The agent can be supervised by the principal or a third party. However, the supervision of healthcare service providers requires that the supervisors also have sufficient healthcare knowledge. Patients generally do not have the ability to supervise, and the supervision needs to be undertaken by a third party. After the establishment of the universal healthcare insurance system, the government as the purchaser of healthcare services naturally needs to undertake the corresponding supervision and restraint tasks. As a result, a dual agency structure has been formed among the government, patients, and hospitals (Wu et al., 2020). The first layer of the principal–agent relationship is formed between patients and the government. Patients pay premiums to the government and entrust the government to select, supervise, and pay for hospitals. There is a second layer of the principal–agent relationship between government and hospitals. Government pays hospitals for healthcare expenses and entrust hospitals to provide effective treatment for patients at a reasonable cost.

In addition, in the context of deepening the reform of HIPM, making full use of big data analysis and information technology to establish scientific and reasonably fee payment standards is conducive in improving the construction of healthcare insurance information systems, establishing a more intelligent information support system, and promoting information sharing between government and hospitals. Meanwhile, it is helpful to establish a mechanism for the efficiency and cost information disclosure of hospitals, to regularly disclose indicators such as costs and patient burden levels, to accept social supervision, to provide a reference for the insured’s choice of healthcare treatment, and to reduce the asymmetry of healthcare information, effectively restrain the overtreatment, and improve QPH.

#### Improving Governance Capability of Government and Reducing Transaction Cost

The new institutional economy theory proposed that there are a series of transaction costs surrounding the signing and implementation of the contract, such as expenditures for searching for information, negotiating, monitoring, and verifying. Williamson (1985) divided them into pre-event and post-event parts. Zhang (1999) indicated that a society with more than one person needs the institution to constrain. Transaction costs are institutional costs. Rearranging the institution will help reduce transaction costs. The empirical research shows that gradual institutional reform can improve the governance ability of the government, reduce transaction costs, and promote economic growth (Xia and Liu, 2017).

Global budget refers to that government pays a fixed fee to the hospital, and the hospital provides patients with healthcare services stipulated in the contract during the contract period (usually one year) (Guo and Gu, 2017). A prospective payment system means that payments occur before healthcare services. Hospitals fully launched open and equal negotiation in advance to determine healthcare service responsibilities and payment rules in the form of contracts. Hospitals are responsible for any excess of the budget (Gu, 2012). The GBPRG transforms the in-process and after-event supervision and verification into the prospective payment system and fixes it in the payment rules, reducing the transaction cost of a large number of verification work in the post-payment system, which is conducive to optimizing the allocation of healthcare resources and promoting the improvement of the QPH.

#### Standardizing Healthcare Service Behavior and Improving Incentive Mechanism

Under the GBPRG, as GB has already reflected the government’s understanding of reasonable expenses for treatment methods, hospitals have to bear the excess part due to excessive healthcare treatment. Therefore, hospitals are endowed with cost control in the new healthcare insurance payment contract, which has the motivation to eliminate overtreatment and strive to adopt the cost minimization strategy to obtain a greater balance. From the perspective of the game, using the rational economic man hypothesis, we can conclude that hospitals need to strengthen the internal system management, optimize the process, improve the incentive, and realize the balance to achieve the Nash equilibrium with healthcare institutions for budget (Du, 2017).

Therefore, the hospital began internal self-revolution in the face of the GBPRG. The strategies for hospitals to deal with GBPRG mainly include controlling hospital healthcare costs, reducing overtreatment, focusing on preventive healthcare services, and changing from the pursuit of service volume to the health status of patients (Shen and Zhang, 2018). Specifically, hospitals will actively control costs and improve the health of patients through a variety of methods (Zhao, 2012). In the internal management, hospitals implement refined cost accounting management, decompose health insurance indicators, improve electronic information systems, and optimize incentives to achieve cost management (Wang, 2008). As for adjustment of service object structure, hospitals need to diversify hospital patient groups, attract more self-paid patients, increase the number of self-paid drugs, and adjust service type structure to reduce the financial risk caused by GBPRG. In addition, adjustment of the healthcare service structure and reduction of the healthcare service has promoted the construction of a hierarchical healthcare system and healthcare alliance (Liu, 2016; Su and Liu, 2021), thereby improving the QPH.

**FIGURE 1:**
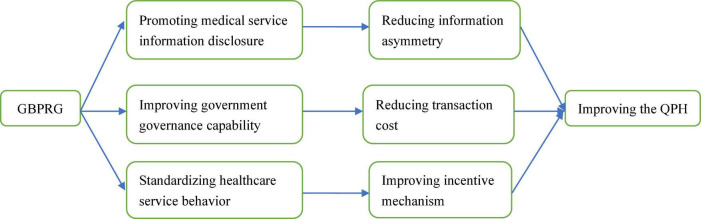
The influence mechanism of global budget payment reform of government (GBPRG) on quality of public healthcare (QPH).

Based on the above analysis, the influence mechanism of GBPRG on QPH is listed in Figure 1.

## Research Design

### Data Sources

To evaluate the impact of GBPR on QPH, we used computer science and big data technology to match the healthcare insurance settlement data with healthcare record data to form comprehensive big data of healthcare and finance exactly. This is an important exploration and innovation in data, which is first time reported in the related research.

We used the large sample data of urban employee healthcare insurance reimbursement settlement from 2012 to 2014 provided by the Chengdu Healthcare Security Administration. After grouping and matching exactly, there are 519,471 matched records, which mainly include healthcare record homepage information (disease diagnosis, ICD-10 code, outcome, hospital level, etc.), healthcare expense settlement information (actual compensation, total healthcare expenses, drug expenses, inspection expenses, material expenses, nursing expenses, bed expenses, treatment expenses, surgical expenses, etc.), and the basic characteristics of patients (age, gender, length of stay, etc.).

Global budget payment reform of government was started in all Grade II Level hospitals in Chengdu on July 1, 2013. To identify the net effect of GBPRG and not be affected by other HIPM, we take July 1, 2013 as the time point of GBPRG in Chengdu, and the selected data time range is from January 1, 2012 to December 31, 2014, which covers before and after GBPRG. According to the HIPM in Chengdu, acute suppurative appendicitis was paid by diagnosis diseases groups (DRGS) and acute appendicitis was paid by GB. So we select acute suppurative appendicitis as the experimental group and acute appendicitis as the control group. As shown in Table 1, acute suppurative appendicitis paid by DRGS and acute appendicitis paid by GB were 4,432 matched records.

**TABLE 1:** Comparison of ICD-10 codes and disease names of global budget payment reform of government (GBPRG) and non-GBPRG.

Classification	Disease name	ICD-10 code	Number
GBPRG	Acute suppurative appendicitis	K35.101	2876
Non-GBPRG	Acute appendicitis	K35.902	1556
Total			4432

We analyze the impact of the GBPRG on QPH, which is mainly manifested in the effect of GBPRG on the outcome of healthcare. The DID method can identify the policy effect that are not affected by GBPRG and eliminate the effects that have not been affected by GBPRG, so that the policy impact effect from GBPRG can be reflected, and the effect on QPH can be evaluated. Therefore, we used the quasi-natural experiment of the GBPRG of Chengdu and DID method to eliminate the effects of other factors affecting the QPH, and estimated the relative pure treatment effect of the GBPRG on QPH.

The dependent variables were the healthcare outcome of patients. The outcome of patients represents the medical result of the patient after seeing a doctor, which can reflect the medical effect of the medical institution to a certain extent, including death, transfer, and rehabilitation. According to existing researches, we used the results of patients’ discharge as a measure of QPH, which is relatively intuitive and can be used in empirical analysis in the measurement of QPH (Yu et al., 2013; Li and Yu, 2019). The control variables mainly were the characteristics of patients, including age, gender, hospital level, healthcare expense structure, etc. Healthcare expenses mainly include total healthcare expenses, inspection expenses, medicine expenses, nursing expenses, bed expenses, material expenses, etc.

### Model Setting

In many existing studies from home and abroad, DID is a widely used method for evaluating policy effects (Card and Krueger, 1993; Duflo, 2001; Zhang et al., 2014; Chen et al., 2018). According to the DID method used to evaluate the effect of policies (He and Shen, 2020; Xiang et al., 2020), the model is set as follows:


(1) $$Y_{i\,t}=\beta_{0}+\beta_{1}\,T_{i}+\beta_{2}\,G_{i}+\beta_{3}\,T_{i}\times G_{i}+\gamma \,\mu_{i\,t}+\varepsilon_{i\,t}$$

*Y*_*it*_ is the dependent variable, mainly including healthcare outcome of patients (death, transfer, and rehabilitation). *T*_*i*_ is the time dummy variable, *T_*i*_* = 0 means before GBPRG, *T_*i*_* = 1 means after GBPRG. *G*_*i*_ is the policy change dummy variable, *G_*i*_* = 1 means the experimental group (acute suppurative appendicitis), *G_*i*_* = 0 means the control group (acute appendicitis). μ_*it*_ is the control variable, including three groups of variables. The first group of variables is the basic characteristics of patients (age, gender, length of stay, etc.), the second group of variables is the hospital level dummy variable, and the third group of variables is the structure of healthcare expenses (total healthcare expenses, drug expenses, inspection expenses, material expenses, etc.).

## Results

### Descriptive Results

From Table 2, we found the basic characteristics of patients. A total of 53% of the patients are women, with an average age of 71.98 years. The hospitals are mainly concentrated in Grade II Level A hospitals and Grade II Level B hospitals. Some patients recovered and left the hospital with an average length stay of 6.15 days. All expenses variables in the study are converted to 2012 yuan using the Consumer Price Index (CPI).

**TABLE 2:** The basic characteristics of patients.

Variable name	Full sample		In 2012		In 2013		In 2014	
	Mean	Std. dev.	Mean	Std. dev.	Mean	Std. dev.	Mean	Std. dev.
Gender	0.53	0.50	0.55	0.50	0.52	0.50	0.51	0.50
Age	71.98	21.33	72.54	21.43	71.11	21.42	72.22	21.17
Hospital level	2.52	1.07	2.44	1.13	2.52	1.06	2.58	1.04
Outcome of patients	2.93	0.25	2.93	0.26	2.93	0.26	2.94	0.24
Length of stay	6.15	3.03	6.27	2.92	6.23	3.43	6.01	2.78
Number	4432		1307		1338		1787	

From Table 3, we found that the average total healthcare expenses from 2012 to 2014 were 4,590 Yuan (CNY) (USD $655.7), 4,840 Yuan (CNY) (USD $691.4), and 5,140 Yuan (CNY) (USD $734.3), respectively. The average drug expenses from 2012 to 2014 were 1,740 Yuan (CNY) (USD $248.6), 1,650 Yuan (CNY) (USD $235.7), and 1,660 Yuan (CNY) (USD $237.1), accounting for 37.91, 34.09, and 32.30% of the total healthcare expenses, respectively. The drug expenses accounted for the total medical expenses continue to decline. However, from 2012 to 2014, the average inspection expenses were 930 Yuan (CNY) (USD $132.9), 1,070 Yuan (CNY) (USD $152.9), and 1,220 Yuan (CNY) (USD $174.3), and the average material expenses were 540 Yuan (CNY) (USD $77.1), 630 Yuan (CNY) (USD $90.0), and 680 Yuan (CNY) (USD $97.1), respectively. The average inspection expenses and material expenses were all increased. At the same time, from 2012 to 2014, the average actual compensation expenses were 2,830 Yuan (CNY) (USD $404.3), 2,980 Yuan (CNY) (USD $425.7), and 3,130 Yuan (CNY) (USD $447.1), and the average reimbursement ratios were 61, 61, and 60%, and the personal expenses were 1,760 Yuan (CNY) (USD $251.4), 1,860 Yuan (CNY) (USD $265.7), 2,020 Yuan (CNY) (USD $288.6), respectively. It was indicated that the actual compensation expenses of healthcare insurance increased, and the reimbursement ratio remained basically the same, but the personal expenses gradually increased.

**TABLE 3:** Descriptive results of the main variables.

Variable name	Full sample		GBPRG		Non-GBPRG	
	Mean	Std. dev.	Mean	Std. dev.	Mean	Std. dev.
**A: In 2012**						
Total healthcare expenses	4.59	2.13	5.33	1.80	3.18	1.98
Drug expenses	1.74	1.05	1.96	1.02	1.32	0.98
Inspection expenses	0.93	0.43	0.98	0.39	0.83	0.49
Material expenses	0.54	0.09	0.70	0.50	0.24	0.38
Nursing expenses	0.08	0.51	0.09	0.05	0.07	0.05
Bed expenses	0.15	0.09	0.16	0.09	0.13	0.09
Surgical expenses	0.76	0.49	0.99	0.29	0.30	0.46
Treatment expenses	0.24	0.16	0.27	0.15	0.17	0.15
Reimbursement ratio	0.61	0.11	0.62	0.12	0.60	0.11
Personal expenses	1.76	1.01	2.05	0.98	1.21	0.82
Actual compensation	2.83	1.45	3.28	1.28	1.97	1.36
Number	1307		860		447	
**B: In 2013**						
Total healthcare expenses	4.84	2.69	5.69	2.17	3.36	2.87
Drug expenses	1.65	1.19	1.87	1.16	1.26	1.14
Inspection expenses	1.07	0.60	1.13	0.55	0.97	0.66
Material expenses	0.63	0.56	0.82	0.52	0.30	0.47
Nursing expenses	0.12	0.07	0.13	0.06	0.11	0.10
Bed expenses	0.14	0.10	0.15	0.08	0.13	0.12
Surgical expenses	0.74	0.53	1.03	0.34	0.25	0.45
Treatment expenses	0.26	0.43	0.30	0.21	0.20	0.66
Reimbursement ratio	0.61	0.12	0.61	0.11	0.61	0.13
Personal expenses	1.86	1.19	2.20	0.98	1.28	1.31
Actual compensation	2.98	1.83	3.50	1.64	2.08	1.79
Number	1338		851		487	
**C: In 2014**						
Total healthcare expenses	5.14	2.66	6.11	2.48	3.34	1.94
Drug expenses	1.66	1.17	1.93	1.24	1.16	0.83
Inspection expenses	1.22	0.67	1.29	0.69	1.08	0.62
Material expenses	0.68	0.61	0.90	0.60	0.26	0.36
Nursing expenses	0.12	0.08	0.14	0.08	0.10	0.07
Bed expenses	0.13	0.08	0.15	0.08	0.11	0.07
Surgical expenses	0.80	0.59	1.08	0.39	0.27	0.53
Treatment expenses	0.29	0.28	0.35	0.31	0.19	0.18
Reimbursement ratio	0.60	0.12	0.60	0.12	0.61	0.12
Personal expenses	2.02	1.16	2.43	1.09	1.25	0.88
Actual compensation	3.13	1.91	3.68	1.94	2.09	1.33
Number	1787		1165		662	

The unit of all expenses variables is 1,000 Yuan (CNY), and the unit of reimbursement ratio is %.

### Parallel Trend Test

The DID method used for policy evaluation must ensure that the changes in the outcome variables of the experimental group and the control group before and after the policy have nothing to do with the control group. The effectiveness of DID requires that the outcome variables of the experimental group and the control group have no systematic or statistically significant differences in the absence of policy. As it is impossible to observe counterfactual situations without policy influence, we cannot directly test DID identification hypothesis. At present, the common practice in existing studies is to indirectly test DID identification hypothesis by testing whether the outcome variables of the experimental group and the control group have the same time trend before the policy occurs (pretend test). At present, this is a common practice using DID method at home and abroad (Card and Krueger, 1993; Duflo, 2001; Zhang et al., 2014; Chen et al., 2018).

**FIGURE 2:**
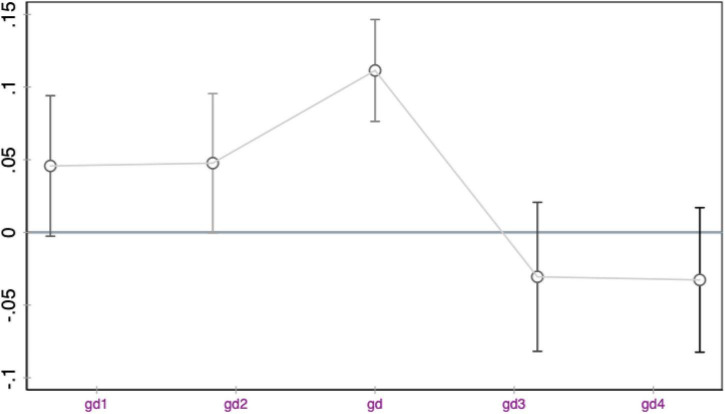
Difference in difference (DID) parallel trend test (healthcare outcome cross-term coefficient).

Before conducting the empirical analysis, we first test the hypothesis. We test the parallel trend hypothesis by changing the product of the experimental group dummy variable and the time dummy variable. The specific method is as follows: We suppose that before adjusting the dummy variable to GBPRG, if the cross-term coefficient obtained by the regression using model (1) is not significant, and after adjusting to GBPRG, the cross-term coefficient obtained by the regression using model (1) is significant. Then, it is consistent with the parallel trend hypothesis. We first divide the data before GBPRG into two groups, assuming that the period from January 1, 2012 to September 30, 2012 is not affected by GBPRG, and from October 1, 2012 to June 30, 2013 is affected by GBPRG. The effect of GBPRG is regressed according to model (1), and the cross-term coefficient gd1 is not significant. Then, we divide the data after GBPRG into two groups, assuming that the period from July 1, 2013 to March 31, 2014 is not affected by GBPRG, and the period from April 1, 2014 to December 31, 2014 is affected by GBPRG. The effect of GBPRG is regressed according to model (1), and the cross-term coefficient gd4 is not significant. Next, we assume that the implementation time of GBPRG is November 1, 2012, the period from January 1, 2012 to October 31, 2012 is not affected by GBPRG, and the period from November 1, 2012 to June 30, 2013 is affected by GBPRG, regression is performed according to model (1), and the cross-term coefficient gd2 is not significant. Finally, it is assumed that the implementation time of GBPRG is April 1, 2014. From July 1, 2013 to March 31, 2014, it is not affected by GBPRG, and it is affected from April 1, 2014 to December 31, 2014. The policy impact of GBPRG is regressed according to model (1), and the cross-term coefficient gd3 is not significant. From Figure 2, we can find that the results of the parallel trend test on the healthcare outcome results support the parallel trend hypothesis.

### Regression Analysis

From Table 4, we can find the cross-term coefficient of T and G represents the effect of GBPRG on QPH. From an overall point of view, after the implementation of GBPRG, the healthcare outcome increased significantly by 12.3%, indicating that GBPRG has a significant positive impact on the healthcare outcome and has a significant effect on improving QPH. From the perspective of the healthcare outcome structure, after GBPRG, death was significantly reduced by 7.1%, transfer was significantly reduced by 5.0%, and rehabilitation was significantly increased by 12.1%, indicating that after GBPRG, deaths and transfers have decreased significantly, rehabilitation has increased significantly, and QPH has improved significantly.

**TABLE 4:** The impact of the global budget payment reform of government (GBPRG) on quality of public healthcare (QPH).

Variable	Healthcare outcome	Death	Transfer	Rehabilitation
T	–0.032** (0.016)	–0.016 (0.012)	0.046*** (0.011)	–0.030* (0.016)
G	0.091*** (0.016)	–0.087*** (0.014)	–0.004 (0.006)	0.091*** (0.016)
T*G	0.123*** (0.018)	–0.071*** (0.013)	–0.050*** (0.013)	0.121*** (0.018)
Gender	0.001 (0.007)	0.000 (0.005)	–0.003 (0.005)	–0.171*** (0.018)
Age	–0.014 (0.013)	0.026*** (0.010)	–0.012 (0.009)	0.002 (0.007)
Length of stay	0.086*** (0.022)	–0.070*** (0.019)	–0.014 (0.012)	0.084*** (0.021)
Total healthcare expenses	–0.867*** (0.245)	0.709*** (0.199)	0.154* (0.082)	–0.863*** (0.245)
Drug expenses	0.018 (0.015)	–0.034** (0.014)	0.016 (0.008)	0.018 (0.015)
Inspection expenses	0.023* (0.013)	–0.008 (0.009)	–0.014 (0.009)	0.022* (0.013)
Material expenses	0.014* (0.008)	–0.014*** (0.005)	–0.029*** (0.006)	0.015** (0.008)
Bed expenses	0.025*** (0.009)	–0.016** (0.007)	–0.009 (0.006)	0.025*** (0.009)
Surgical expenses	0.001 (0.003)	–0.007*** (0.003)	–0.007*** (0.002)	0.000 (0.003)
Treatment expenses	0.049*** (0.009)	–0.031*** (0.008)	–0.016*** (0.006)	0.048*** (0.009)
Actual compensation	0.546 (0.340)	–0.896*** (0.263)	0.351** (0.150)	0.545 (0.341)
Personal expenses	0.155 (0.128)	0.301*** (0.071)	–0.454*** (0.096)	0.153 (0.128)
Reimbursement ratio	–0.688 (0.880)	2.413*** (0.616)	–1.722*** (0.495)	–0.691 (0.881)
Hospital level	Control	Control	Control	Control
*N*	4332	4332	4332	4332
*R* ^2^	0.11	0.14	0.12	0.11

Healthcare expenses are all logarithms; The robust standard errors of clustering at the individual level are in parentheses; *** p < 0.01; ** p < 0.05; * p < 0.1.

From the perspective of the characteristics of patients, gender has no significant effect on the healthcare outcome, death, and transfer, but gender has a significantly negative impact on rehabilitation, indicating that men’s rehabilitation is better. The effect of age on the healthcare outcome, transfer, and rehabilitation are not significant, but the effect of age on rehabilitation is significantly positive, indicating that the older the age, the higher the probability of death. The length of stay has a significant positive impact on the healthcare outcome and rehabilitation and a significant negative impact on death, indicating that the more length of stay, the lower the probability of death and the higher possibility of recovery, and the higher the QPH. The total healthcare expenses have a significantly negative impact on the healthcare outcome and rehabilitation, indicating that it is not that the higher the total healthcare expenses, the better the QPH.

From the perspective of the structure of healthcare expenses, the influence of the structure of healthcare expenses on the healthcare outcome is not consistent. The impact of drug expenses on the healthcare outcome, transfer and rehabilitation is positive, but not significant, and the impact on death is significantly negative, indicating that the higher the drug expenses, the lower the probability of death, which may be related to medicine can reduce the likelihood of patient death. The impact of inspection expenses on the healthcare outcome and rehabilitation is significantly positive, and the impact on death and transfer is negative, but not significant, indicating that the higher the inspection expenses, the lower the probability of death and transfer, and the higher possibility of recovery, which may be related to the detection of diseases in advance for prevention. The impact of material expenses on death and transfer was significantly negative, indicating that the higher the material expenses, the lower the possibility of death and transfer. This may be because hospitals use more medical materials for patients to improve QPH. The impact of bed expenses on the healthcare outcome and rehabilitation is positive, and the impact on death and transfer is significantly negative, indicating that the higher the bed cost, the lower the probability of death and transfer, and the higher the possibility of recovery. This may be related to the length of stay. The longer the length of stay, the better the recovery for patients and the higher the QPH. The surgery expenses have a positive impact on the healthcare outcome and rehabilitation, and a significant negative impact on death and transfer, indicating that the higher the surgery expenses, the lower the probability of death and transfer, and the higher the probability of recovery. The impact of treatment expenses on the healthcare outcome and rehabilitation was significantly positive, and the impact on death and transfer was significantly negative, indicating that the higher the treatment expenses, the lower the probability of death and transfer, and the higher the probability of recovery.

In terms of healthcare insurance compensation and personal expenses, the actual compensation of healthcare insurance has a significantly negative impact on death, indicating that the higher the actual compensation of healthcare insurance, the lower the probability of death and the higher the QPH. The impact of personal expenses on death was significantly positive, indicating that the higher the personal expenses, the higher the probability of death. This may be related to the personal financial burden. The more the personal expenses are, the patient will give up treatment to increase the likelihood of death. The reimbursement ratio has a significant positive impact on death, indicating that it is not that the higher the reimbursement ratio, the lower the probability of death, and the higher the QPH.

In summary, after GBPRG, the healthcare outcome is significantly improved, the possibility of recovery is increased, and the possibility of death and transfer is reduced. However, we found that: (1) It is not that the higher the healthcare expenses, the higher the QPH. (2) It is not that the higher the reimbursement ratio, the higher the QPH. (3) The government needs to balance the relationship between personal expenses and healthcare insurance reimbursement, optimize the allocation of healthcare resources, and realize the pareto optimal choice.

### Robustness Test

We use the method of virtual policy impact time to test the robustness of the result of DID method. As with the parallel trend test conducted as discussed in the previous section, by using a virtual policy impact time, it can be supported that the relevant result is caused by GBPRG.

The first method moves the virtual policy impact forward. We selected samples from January 1, 2012 to June 30, 2013. According to GBPRG, Chengdu did not implement GBPRG before July 1, 2013. The DID model (2) is used to test whether there is a significant change in the impact of GBPRG on QPH before and after October 1, 2012. Because there was no impact from policy reforms around October 1, 2012, if the effect of GBPRG is stable, there should be no statistically significant difference in QPH before and after October 1, 2012.

The second method shifts the impact of virtual policy back. We select samples from July 1, 2013 to December 31, 2014. According to the GBPRG, Chengdu did not implement GBPRG after July 1, 2013. The DID model (2) is used to test whether there is a significant change in the impact of GBPRG on QPH before and after April 1, 2014. Because there was no impact from policy reforms around April 1, 2014, if the effect of GBPRG is stable, there should be no statistically significant difference in QPH before and after April 1, 2014.

Specifically, according to the previous research (Zhang et al., 2014; Chen et al., 2018), we set up a test model similar to the empirical model (1):


(2) $$Y_{i\,t}=\alpha_{0}+\alpha_{1}\,T_{i}+\alpha_{2}\,G_{i}+\alpha_{3}\,T_{i}\times G_{i}+\gamma \,\mu_{i\,t}+\varepsilon_{i\,t}$$

*Y*_*it*_ is the dependent variable, mainly including healthcare outcome of patients (death, transfer, and rehabilitation). *T*_*i*_ is the time dummy variable, *T_*i*_* = 0 means before GBPRG, *T_*i*_* = 1 means after the GBPRG. *G*_*i*_ is the policy change dummy variable, *G_*i*_* = 1 means the experimental group (acute suppurative appendicitis), *G_*i*_* = 0 means the control group (acute appendicitis). μ_*it*_ is the control variable, including three groups of variables. The first group of variables is the basic characteristics of patients (age, gender, length of stay, etc.), the second group of variables is the hospital level dummy variable, and the third group of variables is the structure of healthcare expenses (total healthcare expenses, drug expenses, inspection expenses, material expenses, etc.).

The difference between the test model (2) and the empirical model (1) is that if GBPRG occurs on October 1, 2012 or after April 1, 2014, *Ti* = 1, otherwise, *Ti* = 0. It is assumed that the GBPRG of Chengdu is on October 1, 2012 or April 1, 2014. Obviously, this is a virtual policy reform shock. If there is no statistically significant difference in the parameters, it means that there is no statistically significant difference in QPH before and after October 1, 2012 or April 1, 2014, which means it has passed the robustness test. From Table 5, it can be found that regardless of whether the virtual policy shock is moved forward or the virtual policy shock is moved backward, death, transfer, and rehabilitation are not significant, indicating that there is no statistically significant difference in QPH, which proves the results of the model is robust.

**TABLE 5:** Robustness test: the impact of virtual policy shocks on quality of public healthcare (QPH).

Time	Virtual policy shock moves forward			Virtual policy shock shifts back		
Variable	Death	Transfer	Rehabilitation	Death	Transfer	Rehabilitation
T*G	0.025 (0.019)	0.012 (0.028)	–0.037 (0.033)	–0.000 (0.021)	0.032 (0.014)	–0.032 (0.025)
Hospital level	Control	Control	Control	Control	Control	Control
Number	1941	1941	1941	2491	2491	2491
*R* ^2^	0.08	0.14	0.13	0.13	0.07	0.12

Healthcare expenses are all logarithms; The robust standard errors of clustering at the individual level in parentheses.

## Discussion

### Mechanism Analysis

Global budget payment reform of government refers to the calculation of the total annual overall compensation and control amount in a certain area based on the number of insured persons, the total number of visits per year, and the level of average consultation expenses per visit, which are used for budget control, contract usage, and over-expenses sharing.

First, from Tables 3, 4, we find that although GBPRG can effectively control the unreasonable growth of healthcare expenses, its influence on the structure of healthcare expenses is inconsistent, and the impact of the structure of healthcare expenses on QPH is also different. This is conducive to promoting the disclosure of healthcare service project information to a certain extent. In the field of healthcare services with serious information asymmetry, healthcare insurance is a third party, and its payment method is the key to solving the problem of information asymmetry. The HIPM has changed from a retrospective to a prospective payment system. Under the GBPRG, hospitals conduct the total number of insured persons in a certain area, the total number of annual consultations, the average consultation expenses level, and the estimated annual overall compensation control total publicity. Hospitals will also disclose healthcare service items, prices, and other information to promote the openness and transparency of healthcare service information. Patients can learn about healthcare insurance compensation information and healthcare service information in a timely manner, reducing information asymmetry gaps between healthcare insurance, hospitals, and patients, which promotes the improvement of QPH.

Second, the transaction cost theory put forward that the allocation of market resources requires additional costs. Institutional reforms can improve the governance capabilities of the government, help reduce transaction costs, improve the efficiency of market resource allocation, and promote the economy and society development (Williamson, 1985; Yang and Nie, 2006). From Table 4, we found that the impact of personal expenses on death is significantly positive, indicating that the higher the personal expenses, the higher the probability of death, which means that the government has not assumed the responsibility of providing basic public healthcare services, which is a government failure performance. The HIPM has changed from a retrospective to a prospective payment system. The GBPRG has fully carried out open and equal negotiation and negotiation in advance by the hospital and the healthcare insurance, and determined the healthcare service responsibilities and payment rules in the form of a contract, and paid the expenses, which effectively improved the governance capabilities of the government. The original payment system transforms the in-process and after-event supervision and verification into the pre-payment system and fixes it in the payment rules, reducing the transaction cost of a large number of verification work in the post-payment system, which is conducive to optimizing the allocation of healthcare resources and promoting the improvement of QPH.

Third, as shown in Table 6, our analysis found that the impact of different hospitals on GBPRG is significantly different. The HIPM has changed from retrospective to prospective payment system. The GBPRG has a strong budget constraint on healthcare service providers, which makes up for the problem of inefficient allocation of healthcare resources caused by government failure. At the same time, the benefits of healthcare service providers under GBPRG depend on their own healthcare costs, which can better form an internal incentive mechanism for healthcare service providers, so that hospitals can transform from external incentives that induce healthcare demand to reduce their own healthcare costs. The GBPRG improves the internal incentives of the hospital, standardizes the healthcare service behavior, improves the incentive mechanism of the healthcare service behavior, strengthens the internal management of the hospital, and promotes the improvement of QPH.

**TABLE 6:** The impact on quality of public healthcare (QPH) in different hospital levels.

Hospital level	Variable	Death	Transfer	Rehabilitation
Grade II Level B hospitals with 10% below	T*G	–0.114 (0.106)	–0.030 (0.044)	0.144 (0.113)
	*R* ^2^	0.28	0.19	0.29
Grade II Level B hospitals	T*G	–0.250*** (0.047)	–0.024* (0.013)	0.227*** (0.048)
	*R* ^2^	0.36	0.13	0.17
Grade II Level A hospitals	T*G	–0.008*** (0.010)	–0.056*** (0.014)	0.064*** (0.017)
	*R* ^2^	0.11	0.11	0.12

Healthcare expenses are all logarithms; the robust standard errors of clustering at the individual level in parentheses; *** p < 0.01; * p < 0.1.

### Heterogeneity Analysis

#### Age Structure

**FIGURE 3:**
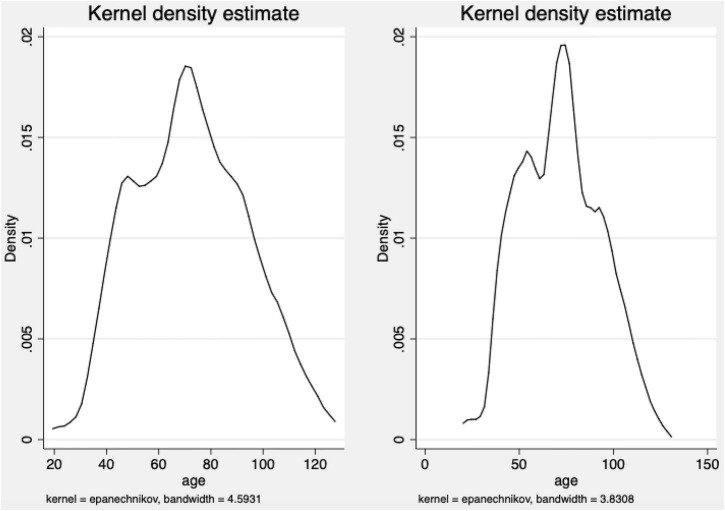
The age distribution density of patients with global budget payment reform of government (GBPRG) and non-GBPRG. The right is the age distribution density of patients with GBPRG, and the left is the age distribution density of patients with non-GBPRG.

The age difference is an important reason for the heterogeneity of GBPRG. From Figure 3, we found that there is a significant difference in the age distribution of patients with GBPRG and non-GBPRG. The age distribution of GBPRG patients is mainly concentrated in 70–90 years old, while the age distribution of non-GBPRG patients is mainly concentrated in 60–80 years old. The age of patients with the GBPRG is obviously older than the age of patients with non-GBPRG. It shows that age difference is an important factor in the heterogeneity of the effect of GBPRG on QPH.

To estimate the effect of GBPRG on QPH for patients of different ages, the model (1) was used to perform DID regression. From Table 7, we found that GBPRG has a significantly negative impact on the death and transfer of the 60–80 years old patients, and the impact on the rehabilitation is significantly positive, indicating the death and transfer of the 60–80 years old patients. The probability is significantly reduced, and the probability of recovery is significantly increased. The GBPRG has no significant effect on QPH for the age groups under 40 and over 100. The effect of GBPRG on QPH for patients of different ages is in line with the age distribution characteristics of GBPRG and non-GBPRG. From the perspective of the effect on QPH, from Table 7, we found that GBPRG has reduced the possibility of death and transfer, and improved QPH.

**TABLE 7:** The impact of quality of public healthcare (QPH) on patients of different ages.

Results	Age < 40	Age < 60	Age < 80	Age < 100	Age < 120
Cross-term coefficient 1 (death)	–0.010 (0.049)	–0.066*** (0.021)	–0.079*** (0.020)	–0.056** (0.027)	–0.054 (0.048)
*R* ^2^	0.13	0.09	0.11	0.13	0.21
Cross-term coefficient 2 (transfer)	–0.060 (0.061)	–0.048** (0.023)	–0.061*** (0.017)	–0.035 (0.026)	–0.024 (0.041)
*R* ^2^	0.09	0.15	0.10	0.12	0.11
Cross-term coefficient 3 (rehabilitation)	0.070 (0.079)	0.114*** (0.031)	0.140*** (0.026)	0.091** (0.037)	0.078 (0.060)
*R* ^2^	0.07	0.09	0.12	0.11	0.20

Healthcare expenses are all logarithms; the robust standard errors of clustering at the individual level in parentheses; ***p < 0.01; **p < 0.05.

#### Hospital Level

For different hospital levels, there may be differences in the impact of GBPRG on QPH. Patients may choose different hospital level for treatment according to factors such as the severity of their own disease, family financial situation, and the distance from the hospital. Therefore, the hospital level is also an important factor in the heterogeneity of the impact of GBPRG on QPH. In order to estimate the heterogeneity of the effect of GBPRG on QPH at different hospital levels, the model (1) is also used for DID regression. From Table 6, we found that for grade II level B hospitals with 10% below, GBPRG has a negative impact on death and transfer, and a positive impact on rehabilitation. For grade II level B hospitals, GBPRG had a significantly negative impact on death, the impact on transfer was significantly negative, and the impact on rehabilitation was significantly positive. For grade II level A hospitals, the impact of GBPRG on death and transfer was significantly negative, and the impact on rehabilitation was significantly positive. It shows that the higher the hospital level, the lower the possibility of death and transfer, the higher the possibility of recovery, and the higher the QPH. The effect of GBPRG on QPH of different hospital levels is quite different. Hospital level is an important factor for the heterogeneity of the effect of GBPRG on QPH.

## Conclusion

We used the large sample data of urban employee healthcare insurance reimbursement settlement from 2012 to 2014 provided by Chengdu Healthcare Security Administration and used DID model to estimate the effect of GBPRG on QPH. We found that after GBPRG, the healthcare outcome is significantly improved, the possibility of recovery is increased, and the possibility of death and transfer is reduced. We found that: (1) It is not that the higher the medical expenses, the higher the QPH. (2) It is not that the higher the reimbursement ratio, the higher the QPH. (3) The government needs to balance the relationship between personal expenses and healthcare insurance reimbursement, optimize the allocation of medical resources, and realize the pareto optimal choice. We analysis of the mechanism and found that after GBPRG, HIPM has changed from retrospective to prospective payment system. By promoting the openness and transparency of healthcare service information, reducing information asymmetry, improving the governance capabilities of the government, reducing transaction costs, improving healthcare service behavior with incentive mechanism, and standardizing healthcare service behavior, GBPRG promoted the improvement of QPH. The results of heterogeneity analysis show that there are significant differences in the effect of GBPRG on QPH of different age groups, especially the most significant effect on the elderly group. Different hospital levels are also important factors for the significant differences in the effect of GBPRG on QPH.

With the development of information technology, HIPM has become an innovative tool for the comprehensive management of healthcare services to improve QPH, and realize the goal of healthy China. The GBPRG is one of the important contents of the reform of HIPM in China. It is the key to the transformation of HIPM from retrospective to prospective payment system. It has played an important role in optimizing the allocation of healthcare resources, regulating the behavior of healthcare services, and improving QPH. The government is mostly concerned about the control of healthcare expenses, but after GBPRG, the changes in QPH especially require the attention of the government. At the same time, the government should continue to explore compound HIPM, improve the governance capabilities of the government, reduce transaction costs, improve healthcare insurance reimbursement policies, adjust the proportion of healthcare insurance reimbursements, continuously optimize the allocation of healthcare resources, establish an incentive mechanism to improve QPH, and realize the pareto optimal choice of healthcare resource allocation.

## Data Availability Statement

The original contributions presented in this study are included in the article/supplementary material, further inquiries can be directed to the corresponding author.

## Author Contributions

LL and SZ contributed to the design and implementation of the research, the analysis of the results, and the writing of the manuscript. Both authors contributed to the article and approved the submitted version.

## Conflict of interest

The authors declare that the research was conducted in the absence of any commercial or financial relationships that could be construed as a potential conflict of interest.

## Publisher’s note

All claims expressed in this article are solely those of the authors and do not necessarily represent those of their affiliated organizations, or those of the publisher, the editors and the reviewers. Any product that may be evaluated in this article, or claim that may be made by its manufacturer, is not guaranteed or endorsed by the publisher.

## References

[B1] Aron-Dine A. Einav L. Finkelstein A. (2013). The Rand health insurance experiment, three decades later. *J. Econ. Perspect.* 27 197–222. 10.1257/jep.27.1.197 24610973 PMC3943162

[B2] Cai Y. Y. Song Y. Wu H. Z. (2013). How to better the medical insurance compensation mechanism in China. *Chin. J. Hosp. Admin.* 29 7–8. 10.3760/cma.j.issn.1000-6672.2013.01.002 30704229

[B3] Card D. Krueger A. B. (1993). Minimum wages and employment: a case study of the fast food industry in New Jersey and Pennsylvania. *Am. Econ. Rev.* 84 772–793. 10.3386/w4509 34419315

[B4] Chang G. M. Cheng S. H. Tung Y. C. (2011). Impact of cuts in reimbursement on outcome of acute myocardial infarction and use of percutaneous coronary intervention a nationwide population-based study over the period 1997 to 2008. *Med. Care* 49 1054–1061. 10.1097/MLR.0b013e318235382b 22009149

[B5] Chen B. Fan V. Y. (2015). Strategic provider behavior under global budget payment with price adjustment in Taiwan. *Health Econ.* 24 1422–1436. 10.1002/hec.3095 25132007 PMC5685661

[B6] Chen Z. Song Z. Zhang C. C. (2018). Effect of separating treatment and drug sales: evidence form medical insurance claims data. *J. Financ. Res.* 10 72–88.

[B7] Cheng S. H. Chen C. C. Tsai S. L. (2012). The impacts of DRGS-based payments on health care provider behaviors under a universal coverage system: a population-based study. *Health Policy* 107 202–208. 10.1016/j.healthpol.2012.03.021 22534586

[B8] Chiu W. H. (1997). Health insurance and the welfare of healthcare consumers. *J. Public Econ.* 64 125–133. 10.1016/S0047-2727(96)01619-2

[B9] Chulis G. S. (1991). Assessing medicare’s prospective payment system for hospitals. *Med. Care Rev.* 48 167–206. 10.1177/002570879104800203 10113662

[B10] Cutler D. M. (1995). The incidence of adverse medical outcomes under prospective payment. *Econometrica* 63 29–50. 10.3386/w4300 34419315

[B11] Cutler D. M. Zeckhauser R. J. (2000). The anatomy of health insurance. *Handb. Health Econ.* 1 563–643. 10.1016/S1574-0064(00)80170-5

[B12] Docteur E. Oxley H. Secretariat O. (2004). *Health System Reform: Lessons from Experience. towards High-Performing Health Systems: Policy Studies.* Paris: Organization for Economic Co-operation and Development.

[B13] Du C. (2017). Dynamic incentive and optimal payment systems for health insurance. *Econ. Res. J.* 52 88–103. 10.1016/j.acra.2011.04.007 21703880

[B14] Duflo E. (2001). Schooling and labor market consequences of school construction in Indonesia: evidence from an unusual policy experiment. *Am. Econ. Rev.* 91 795–813. 10.1257/aer.91.4.795

[B15] Fan V. Y. Savedoff W. D. (2014). The health financing transition: a conceptual framework and empirical evidence. *Soc. Sci. Med.* 105 112–121. 10.1016/j.socscimed.2014.01.014 24524906

[B16] Gao F. (2017). Analysis on the implementation effect of medical insurance expenditure prepayment system. *Chin. Health Econ.* 36 32–34. 10.7664/CHE20171208

[B17] Gu X. (2012). Towards a public contract model: the strategic significance of provider-payment reforms to China’s new healthcare reforms. *Comp. Econ. Soc. Syst.* 4 21–31.

[B18] Guo K. Gu X. (2017). Dealing with the issue of supplier-induced over-consumption: price regulation, competition, or provider-payment reforms? *Soc. Sci. Guangdong* 5 176–185. 10.3969/j.issn.1000-114X.2017.05.021

[B19] Hart O. Holmström B. (1987). “The theory of contracts,” in *Advances in Economic Theory: Fifth World Congress*, ed. Evans D. H. (Boca Raton, FL: CRC Press), 375–405.

[B20] He W. Shen S. G. (2020). Payment schemes of medical insurance and moral hazard of medical service providers—An empirical analysis based on medical insurance reimbursement data. *Stat. Res.* 37 64–76. 10.19343/j.cnki.11-1302/c.2020.08.005

[B21] Holmström B. (1979). Moral hazard and observability. *Bell J. Econ.* 10 74–91. 10.2307/3003320

[B22] Hu J. Zhu X. L. Zheng Y. Dai T. (2019). Study of the effects of the reform on medical insurance payment model in Youxi county of Fujian province. *Chin. J. Health Policy* 12 25–31. 10.3969/j.issn.1674-2982.2019.05.004

[B23] Ji X. R. Wang L. S. Li R. P. (2011). The outpatient prepaying system of total amount in New Rural Cooperative Medical Scheme in Lufeng County Yunnan Province]. *Chin. J. Health Policy* 4 27–33. 10.3969/j.issn.1674-2982.2011.02.007

[B24] Kan K. Li S. F. Tsai W. D. (2014). The impact of global budgeting on treatment intensity and outcomes. *Int. J. Health Care Finance Econ.* 14 311–337. 10.1007/s10754-014-9150-0 25012589

[B25] Lang J. J. Zhou H. L. Yu X. M. (2017). Research on the effects of global budget reform on the cost control of hospitalization patients. *Chin. Health Econ.* 36 20–22. 10.7664/CHE20170205

[B26] Li L. L. Yu Q. (2019). The impact of the reform of the basic medical insurance payment method on medical expenses in China. *Comp. Econ. Soc. Syst.* 2 68–80. 10.1371/journal.pone.0193273 29513712 PMC5841764

[B27] Li S. Q. Chu F. L. (2020). Impact of the reform of the prospective payment system on medical expenses: based on regression discontinuity design. *Chin. Soc. Security Rev.* 4 47–61.

[B28] Liu X. R. (2016). An empirical analysis of the effect of global budget and efforts to increase local community healthcare resource on tiered healthcare system: based on the CHARLS. *Chin. J. Health Policy* 9 16–22. 10.3969/j.issn.1674-2982.2016.04.003

[B29] Liu Y. (2014). Effect on implementation of global budge: based on micro-data of four pilot hospitals in Beijing. *Chin. J. Health Policy* 7 37–42. 10.3969/j.issn.1674-2982.2014.11.007

[B30] Louis D. Z. Yuen E. J. Braga M. Cicchetti A. Rabinowitz C. Laine C. (1999). Impact of a DRGS-based hospital financing system on quality and outcomes of care in Italy. *Health Serv. Res.* 34 405–415. 10199684 PMC1089010

[B31] Ma C. T. A. McGuire T. G. (1997). Optimal health insurance and provider payment. *Am. Econ. Rev.* 87 685–704.

[B32] Miller G. Babiarz K. S. (2014). “Pay-for-performance incentives in low- and middle-income country health programs,” in *Encyclopedia of Health Economics*, ed. Culyer A. J. (San Diego, CA: Elsevier), 457–466. 10.1016/B978-0-12-375678-7.00126-7

[B33] Moreno-Serra R. Wagstaff A. (2010). System-wide impacts of hospital payment reforms: evidence from central and eastern Europe and central Asia. *J. Health Econ.* 29 585–602. 10.1016/j.jhealeco.2010.05.007 20566226

[B34] Mougeot M. Naegelen F. (2005). Hospital price regulation and expenditure cap policy. *J. Health Econ.* 24 55–72. 10.1016/j.jhealeco.2004.04.007 15617788

[B35] Newhouse J. P. (2004). Consumer-directed health plans and the RAND health insurance experiment. *Health Affairs* 23 107–113. 10.1377/hlthaff.23.6.107 15584103

[B36] North D. C. Thomas R. P. (1973). *The Rise of the Western World: A New Economic History.* Cambridge: Cambridge University Press.

[B37] Pauly M. V. McGuire T. G. Barros P. P. (2012). *Handbook of Health Economics.* Amsterdam: North- Holland.

[B38] Shahian D. M. Torchiana D. F. Shemin R. J. Rawn J. D. Normand S. L. (2005). Massachusetts cardiac surgery report card: implications of statistical methodology. *Ann. Thoracic Surg.* 80 2106–2113. 10.1016/j.athoracsur.2005.06.078 16305853

[B39] Shen S. Zhang J. Y. (2018). Transforming and developing health protection: from minimum essential coverage to access to high-quality healthcare. *Chin. Soc. Security Rev.* 2 51–65.

[B40] Su L. Liu Y. Z. (2021). How to achieve the goal of tiering medical services: a study on the health care reform and the interaction between physician and patient in Qingdao. *Hebei Acad. J.* 41 198–206.

[B41] Sun S. X. Sun J. J. Wei J. L. (2012). Effect evaluation of global budget of medical insurance in Beijing. *Chin. Health Econ.* 31 23–25.

[B42] Tung Y. C. Chang G. M. (2010). The effect of cuts in reimbursement on stroke outcome: a nationwide population-based study during the period 1998 to 2007. *Stroke* 41 504–509. 10.1161/strokeaha.109.568956 20075356

[B43] Wang W. Wu C. C. (2017). International experiences and revelation on payment system reform of health insurance. *China Health Insurance* 12 69–72. 10.19546/j.issn.1674-3830.2017.12.018

[B44] Wang Y. Y. (2008). Information asymmetry in medical market: the experience of the United States and its implications for China. *Reform of Economic System* 2 167–170.

[B45] Williamson O. E. (1985). *The Economic Institutions of Capitalism.* New York, NY: Free Press.

[B46] Wolfe P. R. Moran D. W. (1993). Global budgeting in the OECD countries. *Health Care Financ. Rev.* 14 55–76. 10130584 PMC4193373

[B47] Wu S. X. Yang H. Lv H. Q. Yu L. (2020). The principal-agent paradox of corporate governance of public medical institutions and it’s resolving logic. *Zhejiang Acad. J.* 2 97–105. 10.16235/j.cnki.33-1005/c.2020.02.012

[B48] Xia J. C. Liu C. (2017). Administrative approval reform, transaction cost and the economic growth of China. *Manage. World* 4 47–59. 10.3969/j.issn.1002-5502.2017.04.005

[B49] Xiang H. Du C. Peng X. B. (2020). Moral hazard of health insurance based on empirical evidence from compensation policy change. *Insurance Stud.* 6 110–127. 10.13497/j.cnki.is.2020.06.009

[B50] Yang R. L. Nie H. H. (2006). Incomplete contracting theory: a survey. *Econ. Res. J.* 41 104–115.

[B51] Yao Y. Chen Y. Shi J. (2017). Payment reform of medical insurance payment: commentary of international and domestic research progress and China’s practice. *Chin. Health Econ.* 36 36–39. 10.7664/CHE20170409

[B52] Yip W. Eggleston K. (2001). Provider payment reform in China: the case of hospital reimbursement in Hainan province. *Health Econ.* 10 325–339. 10.1002/hec.602 11400255

[B53] Yu Q. Du X. L. Zhao C. W. (2013). On the efficiency of the allocation of limited medical resources across all disease categories. *Soc. Sci. China* 10 61–85.

[B54] Zeng G. H. Jiang C. Z. Wu W. W. (2018). Review on the medical provider behavior under global budget payment]. *Chin. J. Health Policy* 11 8–14. 10.3969/j.issn.1674-2982.2018.09.002

[B55] Zhang C. C. Giles J. Zhao Y. H. (2014). Policy evaluation of China’s new rural pension program: income, property, expenditure, subjective wellbeing and labor supply. *China Econ. Q.* 1 203–230. 10.13821/j.cnki.ceq.2015.01.012

[B56] Zhang M. Ye L. (2014). Analysis and thought of the experiences of medical global budget in pilot Areas. *Chin. Health Econ.* 33 45–47. 10.7664/CHE20141013

[B57] Zhang W. C. (1999). The paradigm of transaction cost. *Soc. Sci. Front* 01 1–9.

[B58] Zhao Y. X. (2012). Introduction of the major payment approaches in Medicare system of USA. *Chin. General Pract.* 15 3321–3322. 10.3969/j.issn.1007-9572.2012.28.038

